# The moral imperative to continue gene editing research on human embryos

**DOI:** 10.1007/s13238-015-0184-y

**Published:** 2015-06-26

**Authors:** Julian Savulescu, Jonathan Pugh, Thomas Douglas, Christopher Gyngell

**Affiliations:** Uehiro Centre for Practical Ethics, University of Oxford, Oxford, OX1 1PT UK; Queensland University of Technology, St Lucia, Brisbane, QLD 4072 Australia

The publication of the first study to use gene editing techniques in human embryos (Liang et al., 2015) has drawn outrage from many in the scientific community. The prestigious scientific journals *Nature* and *Science* have published commentaries which call for this research to be strongly discouraged or halted all together (Lanphier et al., 2015; Baltimore et al., 2015). We believe this should be questioned. There is a moral imperative to continue this research.

Gene editing technologies have enormous potential as a therapeutic tool in the fight against disease. Roughly 6% of all births have a serious birth defect, which is genetic or partly genetic in origin (Christianson et al., 2006). Advanced and precise gene editing techniques could virtually eradicate genetic birth defects, thereby benefiting nearly 8 million children every year. In addition 35% of all deaths are due to chronic diseases, such as cancer and diabetes, in those under 70.^1^ Gene editing could significantly lower this disease burden thereby benefiting billions of people around the world over time. To intentionally refrain from engaging in life-saving research is to be morally responsible for the foreseeable, avoidable deaths of those who could have benefitted (Singer, 1993). Research into gene-editing is not an option, it is a moral necessity.

It might be argued that gene editing is unnecessary because couples at risk of having a child with a genetic disorder can use the existing technology of *in vitro* fertilization (IVF), pre-implantation genetic diagnosis (PGD) and termination of pregnancy (TOP) to ensure that they have a healthy child. Multiple embryos could be created and genetically tested, with only disease-free embryos selected for implantation. This works well for single gene Mendelian disorders like Huntington Disease, cystic fibrosis and thalassaemia. However, the power of PGD for avoiding disease is limited by the number of embryos that can be created. Unless vast numbers of embryos are created, it will not be possible to avoid complex multi-genic disease using PGD. Thus diseases with a polygenic contribution, such as schizophrenia, and common dispositions to diseases will not be addressed by genetic selection without radically increasing the numbers of embryos a couple produces (to hundreds of thousands) (Bourne et al., 2012). Gene editing potentially allows a much wider range of dispositions to disease to be tackled. Furthermore, the power of PGD is also limited by the genetic characteristics of the parents. For instance, in many cases, all embryos produced by a couple will be carriers of the condition that the couple are seeking to avoid passing on. This means that, even if they are able to ‘select’ an unaffected child, the possibility of transmission will arise again at the next generation if a carrier is transferred. By contrast, gene-editing technologies could potentially allow the couple to have a child who does not carry the condition, thus sparing future generations from further gene editing or PGD.

However, some in the scientific community are calling a moratorium on gene editing research. Yet, they fail to give a sufficient justification for such a ban. In calling for a moratorium, Lanphier et al. (2015) state:

*In our view, genome editing in human embryos using current technologies could have unpredictable effects on future generations. This makes it dangerous and ethically unacceptable. Such research could be exploited for non-therapeutic modifications*

This reasoning is, however, inconsistent with widely accepted practices. Nearly all new technologies have unpredictable effects on future generations. Information technologies like the internet and mobile phones fundamentally change the way people interact and communicate with each other. Their effect on future generations is very hard to predict, and though they *could* be catastrophic (for example, through cyberterrorism), this does not mean on balance they should be banned. Their expected benefits outweigh their expected harms.

Another relevant example here is IVF and PGD. PGD requires removing two cells from the 8 cell embryo. It excises 1/4 of the embryo. This could have been much more devastating than gene editing yet it has been proven to be safe. Nevertheless, when introduced it was certainly unpredictable what the effects would be for future generations.

A further reason given as a justification for moratorium on gene editing research is that it will send us on a slippery slope to non-therapeutic modifications and designer babies. But the mere fact that a technology could be used non-therapeutically doesn’t warrant a moratorium on its use. For example, lasik eye surgery can be used non-therapeutically, but this doesn’t justify restrictions on its therapeutic uses. There would need to be clear evidence that the acceptance of therapeutic lasik will ultimately lead to objectionable non-therapeutic applications. Similarly, IVF and PGD can be used to select for traits like height and intelligence. This doesn’t constitute a good reason not to use these technologies to avoid genetic disease. Rather than a blanket ban on research into gene editing technology, it would be more appropriate to ban the deployment of this technology to enhance normal traits, if that is the concern. Technology can and must be controlled by laws. And if it cannot, there is no point in making laws, including bans. Rather, energy would be better spent preparing to combat the unethical deployment of technology.

The clearest ethical concerns regarding current gene editing techniques is that they are unsafe. The study by Huang and co-authors showed that current gene editing techniques can lead to a large number of off-target mutations. This could cause significant defects and disabilities in any individuals born as the result of the research. While some research suggests there are ways to edit genes that greatly reduce the number of off-target mutations (Iyer et al., 2015), it would be highly unethical to bring modified human embryos to term unless we were very confident that the technique could be used safely. The risk would simply not be justified by any potential benefits.

However this doesn’t justify a moratorium on gene editing research. There is already global agreement that no experiments should be conducted where there is a high risk of harm to the participant, and a low chance of benefit. There is already a moratorium on unsafe research and we don’t need a further moratorium on unsafe gene editing research. It is possible to do this research so that the risk is reasonable to any future child resulting from the future use of such techniques therapeutically. As the study by Huang and co-authors shows, much research on gene editing can be conducted now that satisfies global safety guidelines. This research was carried out using tripronuclear (3PN) zygotes, which have one oocyte nucleus and two sperm nuclei. Polyspermic zygotes such as these occur naturally in ~2%–5% of zygotes during *in vitro* fertilization (IVF) clinical trials. Crucially, these zygotes invariably fail to develop normally *in vivo* (Munné and Cohen, 1998), so they are not considered to be viable for implantation. They will never produce a live baby. Since trialling the CRISPR system in these zygotes had no chance of resulting in a live birth, it is unclear how the study could harm or wrong anyone directly. In fact, this research is important precisely because it increases our understanding about some of the risks involved in targeting humans with current gene editing techniques. One of the stated aims of the research was to determine the frequency of off-target effects when CRISPR is used in human embryos. This type of research is important for increasing our understanding of the types of challenges involved in advancing gene editing techniques to the point where they can be used therapeutically.

It should also be acknowledged that the destruction of the tripronuclear zygotes in this research is not morally problematic in the way that the destruction of human embryos in other forms of research is often claimed to be. First, unlike the embryos destroyed in, say, human embryonic stem cell (hESC) research, these zygotes could not have been implanted and developed to term. As such, even for those who ascribe moral status to the embryos used in hESC research on the basis of their potential to develop into a person, it is difficult to see how one could ascribe such status to a tripronuclear zygote on this basis.

Second, the zygotes used in this research were not created for research purposes. In the context of hESC research, a number of bioethicists have objected to the use of embryos that have been created solely for use in this research (President’s Council on Bioethics, 2002; FitzPatrick, 2003; Annas et al., 1996; Outka, 2002). However, many of those who raise such objections agree that it can be morally permissible to use embryos that were created for reproductive purposes, but that are now deemed surplus to requirements for this purpose, and will now be discarded and destroyed. Indeed, the law in some jurisdictions (eg UK and Australia) requires the destruction of such embryos after a certain period of time (normally 10 years) (National Health and Medical Research Council, 2007; The Human Fertilisation and Embryology Authority, 2008). These embryos are in a sense ‘bound to die’, and it is permissible to benefit from their inevitable destruction (Outka, 2002). We believe that there are good arguments for rejecting this moral distinction between the use of created embryos and discarded embryos in hESC research, and that it is morally permissible to carry out research using both created and discarded embryos (Devolder 2005, 2013; Savulescu, 1999; Devolder and Savulescu, 2005). However, one need not endorse this stance in order to believe that the destruction of tripronuclear zygotes in gene-editing research is morally permissible. Even if a tripronuclear zygote has the same moral status as a human embryo, and even if this status rules out the creation of embryos for use in research (both claims we might plausibly deny), it can arguably still be permissible to destroy embryos that were originally created for reproductive purposes but which are no longer needed for that purpose. Many countries already permit hESC research that involves the destruction of such embryos; if anything, the gene-editing research under consideration is less problematic, since there are good reasons to believe that tripronuclear zygotes lack the moral status that embryos are often claimed to have.

Using this principle, embryos from genetic selection, such as those with cystic fibrosis or thalassaemia, that would be destroyed would also be appropriate targets of research. Thus, effects of gene editing on disease mutations could be pursued ethically.

Paradoxically, the most ethical research at this time of uncertainty about management of off target mutations that also explores the potential to combat disease and aging is research with firm endpoints for destruction of embryos, that is, destructive embryo research. In the UK, embryos can be created for research but must be destroyed by 14 days. This kind of time limit would be appropriate for early stage gene editing research. While many object to destructive embryo research on religious grounds, such objections are inconsistent with the existence of IVF involving the production of excess embryos, birth control methods which involve destruction of embryos for fertility control (such as the contraceptive pill and intrauterine devices), and abortion.

In fact, gene-editing technologies might ultimately lead to far fewer embryos being destroyed for reproductive purposes. Currently, if a carrier of a genetic disease wants to have a child that will not be affected by their parent’s condition, the carrier will often choose to undergo IVF and PGD in order to select a non-affected embryo. This practice often involves the creation, and eventual destruction of, a considerable number of surplus unwanted viable embryos. However, this practice would be rendered obsolete by the availability of safe and effective gene-editing technologies; if such technologies became available, carriers of genetic diseases would not have to produce large numbers of surplus embryos which would eventually be destroyed in order to ensure that they could have a child who was not affected by their parent’s genetic disease.

To date, the weight of reasons favours continuing gene editing research, rather than banning it. Those who believe that gene editing research should be banned or discouraged need to explain why this technology needs to be treated differently to other technologies and other reproductive practices. Moreover, they need to explain how the expected risks outweigh the expected benefits, and why the risks cannot be appropriately managed with more specific legislation.

Aging kills 30 million every year and disables many more. Genetic engineering has already produced Methuselah mice that live twice as long (Bartke et al., 2001). Ultimately, gene editing could be used to delay or turn off aging in humans. This would raise profound ethical and social questions about how long humans should live. But it is ethics, not science or law that should decide these answers.

There are clear moral reasons to continue with gene editing research. Advanced gene editing techniques could reduce the global burden of genetic disease and potentially benefit millions worldwide. This research is a moral imperative. It does indeed raise profound ethical issues but these are best addressed with ethical debate and judicious, selective legislation to prevent abuse and premature use of this promising technology.

## Footnotes

1 Chronic diseases and health promotion, World Health Organisation website, http://www.who.int/chp/en/ (accessed 20 May 2015).
